# Thiazole Antibiotics Target FoxM1 and Induce Apoptosis in Human Cancer Cells

**DOI:** 10.1371/journal.pone.0005592

**Published:** 2009-05-18

**Authors:** Uppoor G. Bhat, Marianna Halasi, Andrei L. Gartel

**Affiliations:** 1 Department of Medicine, University of Illinois at Chicago, Chicago, Illinois, United States of America; 2 Department of Biochemistry and Molecular Genetics, University of Illinois at Chicago, Chicago, Illinois, United States of America; 3 Department of Microbiology and Immunology, University of Illinois at Chicago, Chicago, Illinois, United States of America; Ordway Research Institute, United States of America

## Abstract

Forkhead box M1 (FoxM1) oncogenic transcription factor represents an attractive therapeutic target in the fight against cancer, because it is overexpressed in a majority of human tumors. Recently, using a cell-based assay system we identified thiazole antibiotic Siomycin A as an inhibitor of FoxM1 transcriptional activity. Here, we report that structurally similar thiazole antibiotic, thiostrepton also inhibits the transcriptional activity of FoxM1. Furthermore, we found that these thiopeptides did not inhibit the transcriptional activity of other members of the Forkhead family or some non-related transcription factors. Further experiments revealed that thiazole antibiotics also inhibit FoxM1 expression, but not the expression of other members of the Forkhead box family. In addition, we found that the thiazole antibiotics efficiently inhibited the growth and induced potent apoptosis in human cancer cell lines of different origin. Thiopeptide-induced apoptosis correlated with the suppression of FoxM1 expression, while overexpression of FoxM1 partially protected cancer cells from the thiazole antibiotic-mediated cell death. These data suggest that Siomycin A and thiostrepton may specifically target FoxM1 to induce apoptosis in cancer cells and FoxM1 inhibitors/thiazole antibiotics could be potentially developed as novel anticancer drugs against human neoplasia.

## Introduction

Forkhead box M1 (FoxM1) [1], a transcription factor of the Forkhead family [2] is one of the key positive regulators of the cell cycle. Both the expression and the transcriptional activity of FoxM1 is associated with the proliferative state of cells [1]. It is expressed in all embryonic tissues and in proliferating cells of epithelial and mesenchymal origin [3], [4]. FoxM1 plays a role in the development of the nervous system [5] and it is required for hepatoblast differentiation toward biliary epithelial cell lineages [6] and for embryonic development of the pulmonary vasculature [7]. FoxM1 expression is also induced during lung and liver tissue regeneration and repair. The transcriptional activity of FoxM1 depends on oncogenic Ras-MAPK and Sonic Hedgehog pathways [8], [9]. FoxM1 transcriptionally upregulates target genes involved in cell cycle progression and it is critical for G1/S and G2/M transition, and also for the execution of the mitotic program because FoxM1-depleted cells fail to advance beyond the prophase stage of mitosis [10].

While FoxM1 is one of the most overexpressed genes in human solid tumors (reviewed in [11], [12]), its expression is turned off in terminally differentiated, non-dividing cells [1]. FoxM1 is overexpressed in hepatocellular carcinomas [13], pancreatic carcinomas [14], breast cancers [15], [16], non-small cell lung carcinomas [17], anaplastic astrocytomas and glioblastomas [18], basal cell carcinomas [9] and intrahepatic cholangiocarcinomas [19]. Since the function of FoxM1 is inhibited by several tumor suppressors, such as p19-ARF, pRb, p16 and p53 and activated by multiple oncogenic signaling pathways, FoxM1 may be classified as a proto-oncogene. Inhibition of FoxM1 expression by small interfering RNAs [20], [21] or by a peptide containing amino acids 24–46 of p19^ARF^ [22], [23] reduced anchorage-independent cell growth in vitro and delayed liver tumor growth in mice. Similarly, suppression of FoxM1 in pancreatic cancer cells by RNA interference led to the inhibition of their metastatic potential [24]. These studies have demonstrated that FoxM1 is essential for cancer cell viability and its inhibition may hinder the development of cancer, suggesting that targeting FoxM1 by small molecules could represent a new strategy for developing novel anticancer drugs [25], [26], [27], [28].

Previously, using a cell-based screening system developed by our laboratory, we identified a thiopeptide, Siomycin A (NSC-285116) as a potent inhibitor of FoxM1 [25]. In addition, we showed that Siomycin A and another similar thiazole antibiotic, thiostrepton, which has already been approved by the FDA for animal use, inhibit FoxM1 and induce apoptosis in melanoma cells [26], [29]. Here, we demonstrated that thiazole antibiotics, Siomycin A and thiostrepton inhibit FoxM1 transcriptional activity and expression. We also found direct correlation between the suppression of FoxM1 expression and induction of apoptosis by the thiopeptides in different human cancer cell lines. Furthermore, we established that FoxM1 could protect against cell death induced by the thiazole antibiotics, suggesting that these drugs may partially exert their anticancer activity via the suppression of FoxM1.

## Results

**Figure 1:**
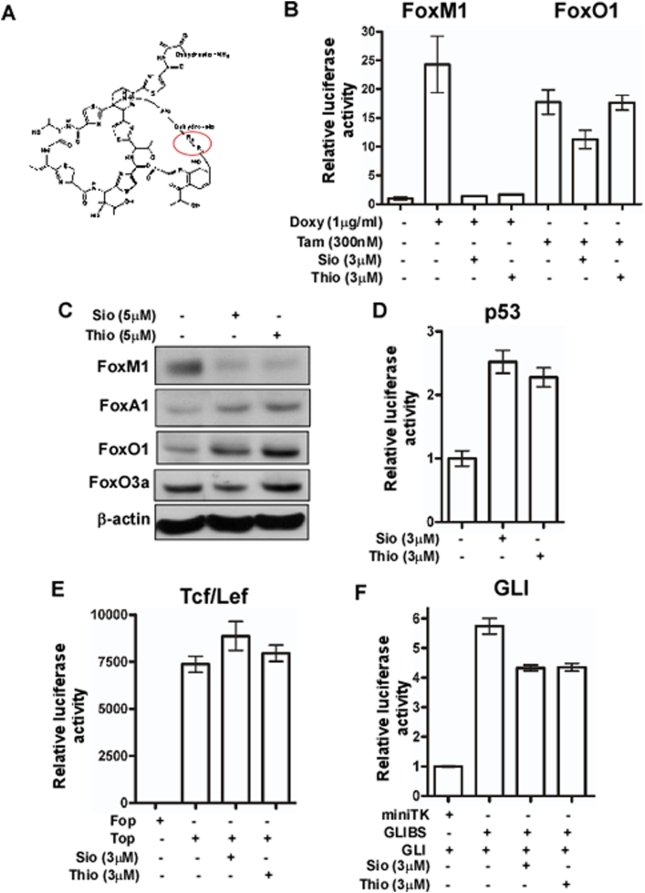
Thiazole antibiotics inhibit FoxM1-dependent transcription and FoxM1 expression. (A) The chemical structure of the thiazole antibiotic, thiostrepton that differs from Siomycin A by only two residues (Thiostrepton-R1-R2: Isoleucine-alanine; Siomycin- R1-R2: valine-dehydroalanine). (B) Luciferase assays after treatment of the C3-Luc2.3-FoxO1 cell line with the combination of either 1 µg/mL doxycycline (Doxy) or 300 nM tamoxifen (Tam) and 3 µM of Siomycin A (Sio) or thiostrepton (Tio), respectively revealed that thiostrepton is also a negative regulator of FoxM1 transcriptional activity and thiazole antibiotics inhibit FoxM1 transcriptional activity among the Forkhead family members. (C) Thiazole antibiotics downregulated FoxM1 protein levels, but not FoxA1, FoxO1 and FoxO3a levels as detected by immunoblotting. (D) The HCT116-p53RE-Luc cell line, which stably expressing firefly luciferase under the control of multiple p53 response elements treated with the indicated concentration of Siomycin A or thiostrepton. After overnight treatment the luciferase activity was measured. (E) SW480 colon cancer cell line was transiently transfected with the Tcf/Lef-dependent TOPFlash and the control FOPFlash constructs. Twenty-four hrs following transfection the cells were treated with 3 µM of Siomycin A or thiostrepton. The next day the luciferase activity was measured. (F) A549 lung cancer cells were transiently transfected with the GLI-dependent GLIBS-Luc, the control miniTK reporter constructs and the GLI expression plasmid. Cells were treated with the indicated concentration of the thiopeptides 24 hrs after transfection and the luciferase activity was measured the following day. Bars in B, D–F are representative mean values of triplicate experiments+/−SD.

Recently, we obtained evidence that another thiazole antibiotic, thiostrepton, which structurally differs from Siomycin A by only 2 residues (Fig. 1A) possesses anti-cancer [30] and anti-FoxM1 properties [29] similar to Siomycin A. To evaluate the effects of thiostrepton on FoxM1 transcriptional activity and also to study how the thiazole antibiotics affect the transcriptional activity of other members of the Forkhead family, we developed the C3-Luc2.3-FoxO1 cell line. C3-Luc2.3-FoxO1 cells are a derivative of U2OS osteosarcoma cells with a doxycycline-inducible FoxM1-GFP fusion protein [25], a tamoxifen-inducible constitutively active FoxO1(AAA)-ER fusion protein and a FoxM1/FoxO1-dependent firefly luciferase. In this system, we were able to selectively induce either FoxM1 transcriptional activity by the addition of doxycycline or FoxO1 transcriptional activity by the addition of tamoxifen. First, to test how thiostrepton affects FoxM1 transcriptional activity compared to Siomycin A, cells were treated with a combination of doxycycline and the thiazole antibiotics and 16 hours later the luciferase activity was measured. We found that the repression of FoxM1 transcriptional activity by thiostrepton is comparable to that of Siomycin A (Fig. 1B), indicating that both thiazole antibiotics inhibit FoxM1 transcriptional activity. Next, to determine whether the thiazole antibiotics inhibit the transcriptional activity of other Forkhead family members such as FoxO1 [31], we treated the C3-Luc2.3-FoxO1 cell line with a combination of tamoxifen and the thiopeptides. We found that addition of tamoxifen led to the induction of FoxO1-dependent luciferase activity, but the treatment with the thiazole antibiotics did not reduce this value (Fig. 1B). Since all members of the Forkhead family share a conserved Forkhead/winged-helix DNA-binding domain that is responsible for binding to consensus sites, our data suggest that the thiopeptides do not target this domain and they may negatively regulate only FoxM1, but not other Forkhead family members.

In our previous reports we found unexpectedly that Siomycin A not only downregulated FoxM1 transcriptional activity, but it also reduced the mRNA and protein levels of FoxM1 [25], [29]. In this study, we demonstrated that thiostrepton also downregulates FoxM1 protein levels to a similar extent to Siomycin A (Fig. 1C). In addition, we found that the thiazole antibiotics did not decrease the protein levels of other members of the Forkhead family such as FoxA1, FoxO1 and FoxO3a (Fig. 1C), further supporting the idea that thiazole antibiotic Siomycin A and thiostrepton are specific inhibitors of FoxM1. To further explore the potential specificity of the antibiotics for FoxM1-dependent transcription, we tested how Siomycin A and thiostrepton affect the transcriptional activity of other transcription factors such as p53, Tcf/Lef and GLI. To this end, the HCT116-p53RE-Luc cell line (with wild-type p53 and multiple p53 response elements upstream of the luciferase gene), SW480 colon cancer cell line (transiently transfected with the Tcf/Lef-dependent TOPFlash construct [32]; a gift from Dr. Randall Moon) and A549 lung cancer cells (transiently transfected with the GLI-dependent GLIBS-Luc reporter construct and the GLI expression plasmid; gifts from Dr. David Robbins) were treated with Siomycin A or thiostrepton and the luciferase activity was measured 16 hours after treatment. We found that treatment with the antibiotics did not reduce p53, Tcf/Lef or GLI- dependent transcription (Fig. 1D–F). However, our further experiments showed that the thiazole antibiotics also affect NF-kB activity, but not the activity of other studied transcription factors (data not shown).

**Figure 2:**
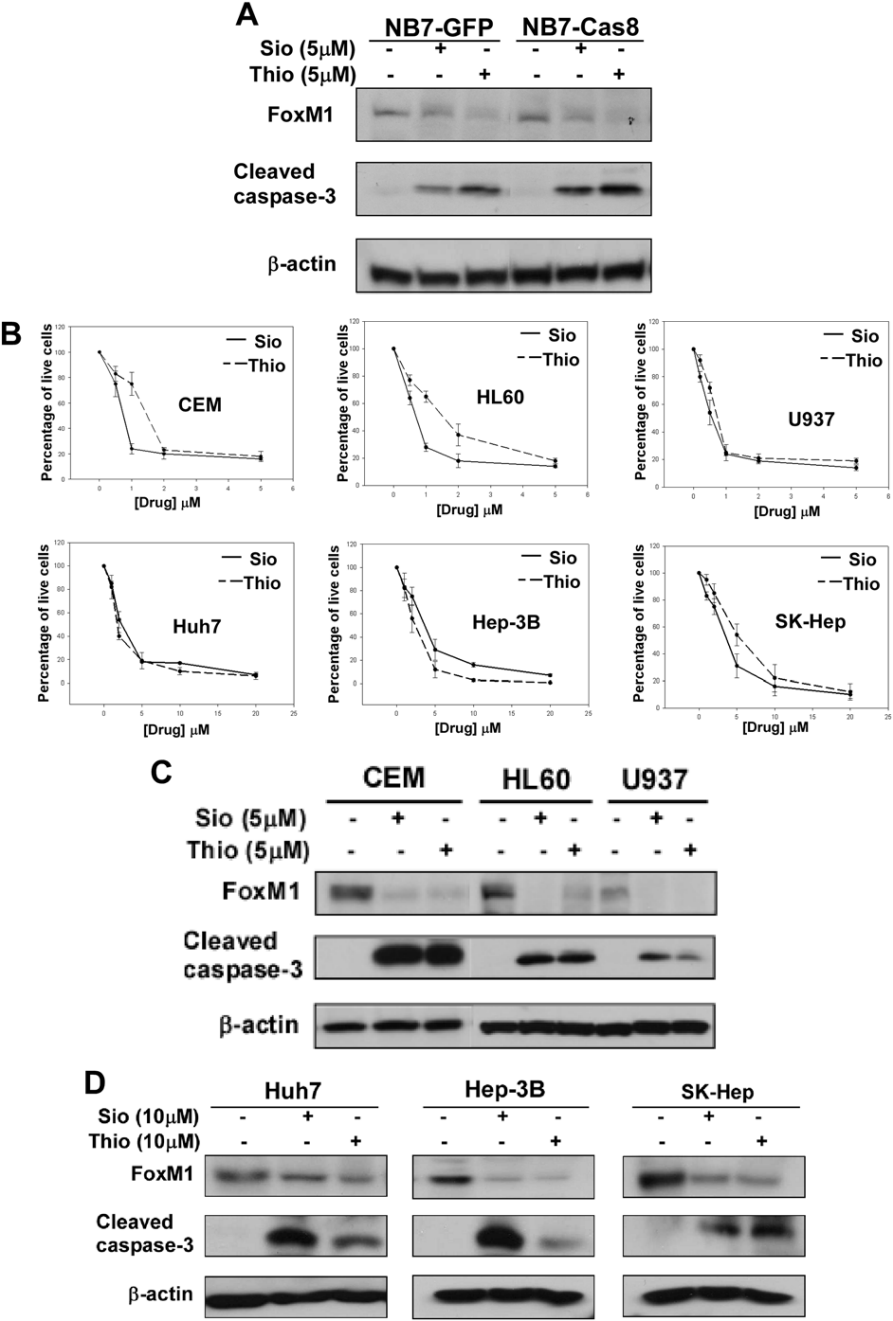
Evaluation of the effects of the thiopeptides on a panel of human cancer cell lines. (A) Treatment of caspase-8 deficient and reconstituted NB7 neuroblastoma cell lines with the thiazole antibiotics revealed that thiopeptide-induced apoptosis mainly involves the intrinsic apoptotic pathway. (B) Leukemia cancer cell lines CEM [IC_50_/µM: Sio-0.73 (0.08); Thio-1.47 (0.1)], HL60 [IC_50_/µM: Sio-0.68 (0.06); Thio-1.78 (0.4)], and U937 [IC_50_/µM: Sio-0.53 (0.1); Thio-0.73 (0.3)], and liver cancer cell lines Hep-3B [IC_50_/µM: Sio-3.6 (1.3); Thio-2.3 (0.8)], Huh7 [IC_50_/µM: Sio-2.3 (0.5); Thio-1.8 (0.2)], and SK-Hep [IC_50_/µM: Sio-3.7 (0.4); Thio-6.0 (1.4)], showed sensitivity in low micromolar range to the thiazole antibiotics as determined by growth inhibition assays. (C–D) Siomycin A and thiostrepton inhibit FoxM1 expression and induce potent apoptosis in leukemia and liver cancer cells.

To evaluate the anticancer potential of the thiazole antibiotics we analyzed their effects on human cancer cell lines of different origin that had elevated expression level of FoxM1. First, we investigated whether the extrinsic or intrinsic apoptotic pathway is involved in the thiopeptide-induced apoptosis. We treated caspase-8 deficient and reconstituted NB7 neuroblastoma cell lines with the antibiotics for 24 hrs (Fig. 2A). We found that caspase-8 deficient NB7 cells that cannot undergo extrinsic apoptosis were almost as sensitive to the thiopeptides as reconstituted NB7 cells with active caspase-8, suggesting that the thiopeptide-induced apoptosis mainly involves the intrinsic apoptotic pathway.

To quantitatively assess the degree of sensitivity of different human cancer cell lines to the thiazole antibiotics Siomycin A and thiostrepton we performed growth inhibition assays on various leukemia (CEM, HL60, U937) and liver (Hep-3B, Huh7, SK-Hep) cancer cells. These cancer cell lines were treated with different concentrations of the antibiotics for 48 hrs and the extent of growth inhibition was determined by counting the number of live cells (Fig. 2B). All of the cell lines displayed IC_50s_ in the low micromolar range, suggesting that these human cancer cells are fairly sensitive to the thiopeptides. In addition, we evaluated the apoptotic response to Siomycin A and thiostrepton in these human cancer cells by immunoblotting for cleaved caspase-3. We found that both Siomycin A and thiostrepton repress FoxM1 protein expression, and induce apoptosis in these leukemia and liver cancer cells (Fig. 2C, D). These data further support our conclusion that thiazole antibiotics not only antagonize the transactivation ability of FoxM1, but they also inhibit its expression because of the FoxM1 positive feedback loop [33]. Furthermore, we observed direct correlation between FoxM1 suppression and caspase-3 cleavage (hallmark of apoptosis) after treatment with these compounds. The close link between FoxM1 repression and induction of apoptosis suggests that the thiopeptides may exert their proapoptotic activity at least partially through the inhibition of FoxM1 in human cancer cells.

**Figure 3:**
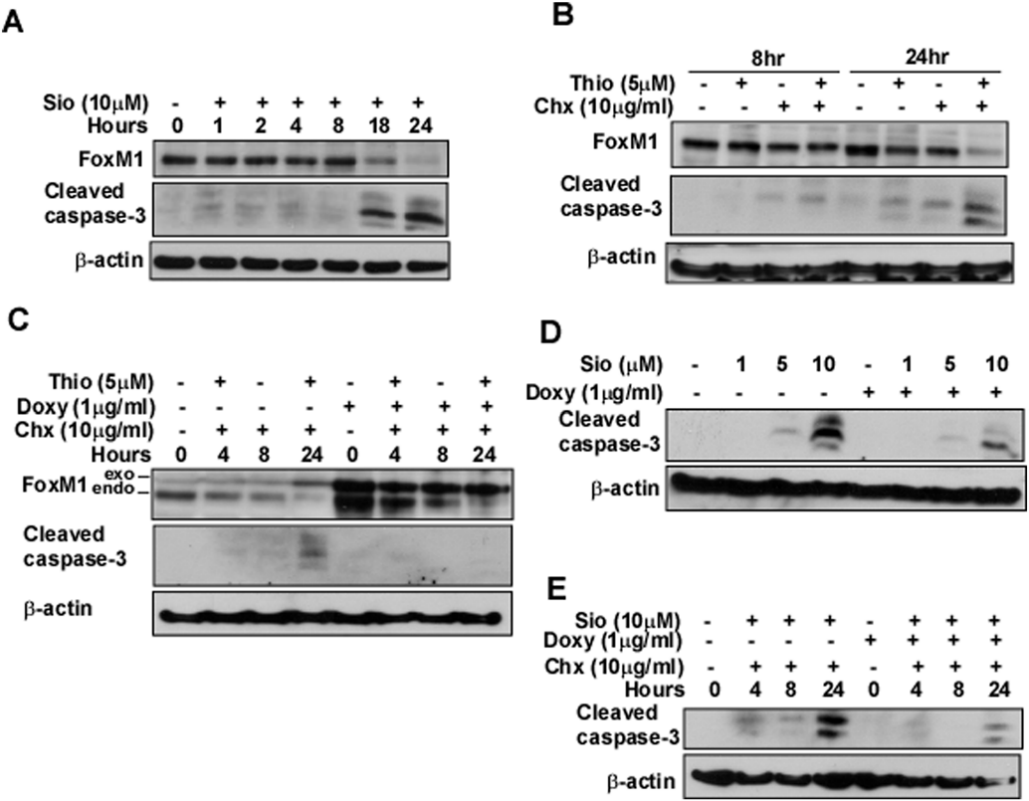
Overexpression of FoxM1 partially protects cancer cell lines from thiazole antibiotic-induced apoptosis. (A) Immunoblot analysis after treatment with Siomycin A revealed close correlation between downregulation of FoxM1 and induction of apoptosis. (B) Following treatment with thiostrepton, thiopeptide-induced apoptosis and inhibition of FoxM1 protein expression are more prominent in the presence of cyclohexamide (Chx) as depicted by immunoblotting for FoxM1 and cleaved caspase-3. (C) The expression of endogenous FoxM1 decreased in a time-dependent fashion in the presence of thiostrepton and Chx, while the levels of exogenous FoxM1 were not affected. Overexpression of FoxM1 protected against cell death induced by thiostrepton as detected by immunoblotting for cleaved caspase-3. (D) FoxM1 overexpressing cells were resistant to the treatment with increasing amount of Siomycin A as analyzed by immunoblotting for cleaved caspase-3. (E) Immunoblot analysis revealed that overexpression of FoxM1 also protected against Siomycin A-induced apoptosis in the presence of Chx.

To investigate the potential role of FoxM1 in the thiopeptide-mediated apoptosis, we treated U2OS osteosarcoma cells with 10 µM of Siomycin A and harvested the cells at different time points (Fig. 3A). We found considerable decrease in FoxM1 protein expression as early as 18 hr, which was correlated with the appearance of robust cleaved caspase-3 bands. We also observed correlation between stronger downregulation of FoxM1 and more intense apoptosis after 24 hr treatment with thiostrepton in the presence of the well-known translational inhibitor cyclohexamide (Chx) compared to individual treatment with the drugs, again indicating that suppression of FoxM1 may be required for the thiopeptide-induced apoptosis (Fig. 3B). To further explore the role of FoxM1 in the thiopeptide-mediated apoptosis we utilized the C3 cell line [25]. FoxM1 expression was induced with the addition of doxycycline and the following day the cells were treated with thiostrepton and cyclohexamide for 24 hours (Fig. 3C). We observed that the expression of endogenous FoxM1 decreased in a time-dependent manner after treatment, while the levels of exogenous FoxM1 were not affected (Fig. 3C). We also found that overexpression of FoxM1 protected against cell death induced by thiostrepton as detected by immunoblotting for cleaved caspase-3 (Fig. 3C). These data support the notion that downregulation of FoxM1 may contribute to the thiopeptide-induced apoptosis. Similarly, we found that C3 cells overexpressing FoxM1 were resistant to the treatment with Siomycin A as analyzed by immunoblotting for cleaved caspase-3 (Fig. 3D). Additional experiments revealed that overexpression of FoxM1 also protects against Siomycin A-induced apoptosis in the presence of cyclohexamide (Fig. 3E). Taken together, all these data suggest that downregulation of FoxM1 by Siomycin A and thiostrepton may be required for the thiopeptide-induced apoptosis.

## Discussion

Previously, we reported that thiazole antibiotics Siomycin A [25] and thiostrepton [29] inhibit FoxM1 and induce apoptosis in human cancer cells. In this study, we demonstrated that the thiazole antibiotics inhibit FoxM1 transcriptional activity, they also downregulate FoxM1 expression and induce cell death in neuroblastoma, leukemia and liver cancer cells. It is known that the thiazole antibiotics carry out their antibacterial activity by inhibiting bacterial translation via interaction with the 23S ribosomal RNA, but they do not block eukaryotic protein synthesis [34]. The precise mechanism of inhibition of FoxM1 transcriptional activity remains to be elucidated, but it is not related to their ability to inhibit protein synthesis. Interestingly, we also found that thiazole antibiotics suppress not only the transcriptional activity, but also the expression of FoxM1 (Fig. 1, 2), suggesting that FoxM1 may positively regulate its own transcription [33].

Here, we found that thiazole antibiotic-induced apoptosis in cancer cells of different origin was correlated with the downregulation of FoxM1 (Fig. 2, 3). The thiopeptides inhibited cell growth with similar IC_50_ and induced cell death with comparable concentrations in such diverse cell types as neuroblastoma, leukemia and hepatoma (Fig. 2). Since we already reported that Siomycin A and thiostrepton target FoxM1, inhibit cell growth and induce apoptosis in melanoma cells [29], these data further confirm that thiazole antibiotics may affect a wide variety of human cancer cells. In addition, we showed that overexpression of FoxM1 could protect cancer cells against thiopeptide-mediated apoptosis (Fig. 3C, D, E). Since thiazole antibiotics suppress the expression and the activity of FoxM1 and at the same time FoxM1 overexpression protects cancer cells from Siomycin A and thiostrepton toxicity, FoxM1 may be a valid target of thiazole antibiotic-induced apoptosis. Recently, Kwok et. al. showed that thiostrepton represses FoxM1 expression and induces apoptosis in breast cancer cells [35]. These data further support our present results and the findings from our previous publications that thiazole antibiotics, Siomycin A [25], and thiostrepton [29] induce apoptosis and suppress FoxM1 expression in human cancer cells. However, this group did not link suppression of FoxM1 expression to inhibition of its transcriptional activity by thiostrepton [35]. In addition, they claim that only constitutively active, but not wildtype FoxM1, may inhibit thiostrepton antiproliferative activity [35]. Further experiments are needed to resolve these differences.

In summary, we demonstrated that thiazole antibiotics Siomycin A and thiostrepton are potent inhibitors of FoxM1 transcriptional activity and expression. In addition, they induce programmed cell death in human cancer cells of diverse origin. The degree of apoptosis induced by the thiopeptides correlates with the suppression of FoxM1, while overexpression of wild type FoxM1 partially protected cancer cells from thiopeptide-induced apoptosis. These data suggest that inhibition of FoxM1 by Siomycin A and thiostrepton to some extent is responsible for the cell death induced by the thiazole antibiotics. The experiments described in this manuscript support our earlier reports [25], [26], [27], [29] that FoxM1 is an appropriate target for anticancer drugs and that thiazole antibiotics could represent promising alternatives to presently used anticancer treatments.

## Materials and Methods

### Cell lines, media and chemical compounds

U2OS osteosarcoma cells; C3 cells [22], a U2OS clone C3 cell line with doxycycline-inducible FoxM1-GFP fusion protein; U2OS derived C3-Luc2.3-FoxO1 osteosarcoma, stably expressing the doxycycline-inducible FoxM1-GFP [25], the tamoxifen-inducible FoxO1(AAA)-ER fusion protein and firefly luciferase under the control of multiple FoxM1/FoxO1 responsive elements; HCT116-p53RE-Luc colon, stably expressing firefly luciferase under the control of a promoter with multiple p53 response elements (p53RE); A549 lung and Huh7, Hep3B and SK-Hep liver cancer cell lines were grown in DMEM medium (Invitrogen). SW480 colon, caspase-8 deficient and reconstituted NB7 neuroblastoma (generous gifts from Dr. Jill M. Lahti, St. Jude Children's Research Hospital, Memphis), CEM, HL60 and U937 leukemia cancer cell lines were grown in RPMI 1640 medium (Invitrogen). In all cases the media were supplemented with 10% fetal bovine serum (Atlanta Biologicals) and 1% penicillin-streptomycin (GIBCO) and the cell lines were kept at 37°C in 5% CO_2_. Thiazole antibiotics Siomycin A (NCI) and thiostrepton (Sigma) were dissolved in DMSO (dimethylsulfoxide), tamoxifen (Sigma) in ethanol, doxycycline (Clontech) in PBS and cyclohexamide (Sigma) in DMSO.

### Constructs and transfections

The Super8xTOPFlash and the control Super8xFOPFlash reporter plasmids were generous gifts from Dr. Randall T Moon (University of Washington, Seattle, WA). The SRαGLI1 expression plasmid and the GLI-BS-Luc, miniTK reporter constructs were kind gifts from Dr. David J. Robbins (Dartmouth Medical School, Hanover, NH). Transient transfections were carried out using Lipofectamine 2000 (Invitrogen) according to the instructions of the manufacturer.

### Immunoblot analysis

Cancer cells of different origin treated as indicated were harvested and lysed by using IP buffer (20 mM HEPES, 1% Triton X-100, 150 mM NaCl, 1 mM EDTA, 1 mM EGTA, 100 mM NaF, 10 mM Na4P2O7, 1 mM sodium othrovanadate, 0.2 mM PMSF supplemented with protease inhibitor tablet (Roche Applied Sciences)). Protein concentration was determined by the Bio-Rad Protein Assay reagent (BIO-RAD). Isolated proteins were separated on 8% or 10% SDS-PAGE and transferred to PVDF membrane (Millipore). Immunoblotting was carried out as described in 34, 35 with antibodies specific for FoxM1 (a gift from Dr. Costa's lab), FoxA1 (a gift from Dr. Costa's lab), FoxO1 (Cell Signaling), FoxO3a (Upstate), Cleaved caspase-3 (Cell signaling) and β-actin (Sigma).

### Luciferase assays

C3-Luc2.3-FoxO1 cells were grown on 6-well plates and treated overnight with the combination of either 1 µg/mL doxycycline or 300 nM tamoxifen and 3 µM of Siomycin A or thiostrepton. Also, the HCT116-p53RE-Luc cell line was grown on 6-well plates and treated with 3 µM of Siomycin A or thiostrepton for 16 hrs. Furthermore, SW480 colon cancer cell line grown on 6-well plates was transiently transfected with the TOPFlash and the FOPFlash constructs. Twenty-four hrs following transfection the cells were treated with 3 µM of Siomycin A or thiostrepton. The next day the firefly luciferase activity was measured using the Luciferase Assay System (Promega). Protein concentration measured by the Bio-Rad Protein Assay reagent (BIO-RAD) was used for normalization. A549 lung cancer cells grown on 6-well plates were transiently cotransfected with either the GLI-dependent GLIBS-Luc or the control miniTK reporter constructs, the GLI expression plasmid and pRL-null (Promega) that expresses renilla luciferase. Cells were treated with 3 µM of Siomycin A or thiostrepton 24 hrs after transfection and the luciferase activity was measured with the Dual-Luciferase Reporter Assay System (Promega) according to the manufacturer's instructions the following day.
